# Astrocytic VPS35 coordinates iron homeostasis with HIF1α-VEGFa signaling to prevent astrocytic ferroptosis and to promote neocortical angiogenesis

**DOI:** 10.1186/s13578-026-01602-2

**Published:** 2026-06-24

**Authors:** Anika Wu, Daehoon Lee, Ananya Nadgauda, Emily Arzola, Lei Xiong, Wen-Cheng Xiong

**Affiliations:** Department of Neurosciences, School of Medicine, Case Western Reserve University, Cleveland, OH 44106 USA

## Abstract

Astrocytes coordinate neuronal activity with vascular development and metabolic homeostasis during postnatal brain maturation, yet the mechanisms enabling astrocytes to support angiogenesis remain elusive. VPS35, a core component of the retromer complex, regulates transmembrane protein trafficking and is implicated in multiple neurodegenerative diseases; however, its function in astrocytes is unknown. Here, we identify astrocytic VPS35 as a crucial regulator for astrocyte maturation and neurovascular development through its control of iron homeostasis and HIF1α–VEGFa signaling. Conditional deletion of Vps35 in astrocytes impaired astrocyte maturation and survival, as well as basal and activity-dependent angiogenesis, thus reducing cerebral blood flow, and inducing widespread vascular abnormalities in both cortical and meningeal vessels. Mechanistically, loss of astrocytic Vps35 resulted in intracellular iron accumulation, likely due to impaired ferroportin (FPN)-mediated iron export. Iron overload enhanced HIF1α hydroxylation and degradation, suppressing VEGFa expression and attenuating angiogenic responses. In parallel, excess iron induced astrocyte ferroptosis, characterized by increased lipid peroxidation, diminished antioxidant capacity, astrocyte loss, and impaired astrocyte maturation. Importantly, iron chelation reduced ferroptotic stress, restored HIF1α-VEGFa signaling, and rescued vascular density in mutant mice. Together, these findings establish astrocytic VPS35 as a critical integrator of endosomal protein trafficking, iron metabolism, and angiogenic signaling, and identify iron dysregulation as a central mechanism linking retromer dysfunction to impaired astrocyte and neurovascular development.

**Supplementary Information:**

The online version contains supplementary material available at https://doi.org/10.1186/s13578-026-01602-2.

## Introduction

Cerebral blood vessels (BVs), composed of endothelial cells (ECs) along with glial and perivascular cells, form a specialized network that includes arteries, capillaries, and veins [1–4]. This vascular system supplies oxygen and nutrients, removes metabolic waste, and regulates cerebral blood flow to meet the brain’s high energy demand [2, 4–6]. In addition, the blood–brain barrier (BBB), established by endothelial tight junctions and supported by astrocytic endfeet, is a selective interface that preserves ionic homeostasis, restricts molecular and immune cell entry into the brain, and protects neural tissue [2, 7–9]. Vascular dysfunction is increasingly recognized as an early and central feature of neurological disorders, including Alzheimer’s disease (AD), AD related dementia (ADRD), Parkinson’s disease (PD), amyotrophic lateral sclerosis (ALS), and stroke [3, 10–12]. Despite distinct etiologies, these conditions share common vascular abnormalities, such as impaired angiogenesis, BBB disruption, and reduced cerebral blood flow, that exacerbate neuronal dysfunction and neurodegeneration [10–12]. Therefore, elucidating the molecular mechanisms underlying cerebral vascular defects in neurodegenerative diseases is of considerable interest.

Astrocytes and cerebral BVs develop in close coordination to establish a functional vascular unit. Cerebral angiogenesis, the sprouting of new vessels from preexisting ones, begins during embryogenesis and continues postnatally to refine and expand the vascular network [13–15]. Astrocyte specification also starts embryonically and extends through the first three postnatal weeks, during which astrocytic processes expand and mature [16, 17]. By postnatal day 14 (P14), astrocytic endfeet contact capillaries, coinciding with BBB maturation [18–20], suggesting a key role for astrocytes in stabilizing the developing vasculature. Astrocytes regulate BV development through both structural and secretory mechanisms: their endfeet ensheathe nearly 90% of the capillary surface, providing physical support and signaling to ECs [4, 8, 21], and they secrete pro-angiogenic factors such as vascular endothelial growth factor a (VEGFa) and transforming growth factor-β (TGF-β), which promote endothelial proliferation, vessel stabilization, and BBB integrity [22–24]. In contrast, under pathological conditions, astrocytes release inflammatory cytokines and reactive oxygen species that impair endothelial function and drive vascular regression [15, 25, 26]. Beyond development, astrocytes are essential for neurovascular coupling, converting neuronal activity into localized changes in blood flow via calcium-dependent signaling that regulates vessel tone [2, 27]. Together, these findings establish astrocytes as central regulators of angiogenesis, cerebral perfusion, and metabolic exchange, although the mechanisms coordinating astrocytic vascular signaling and metabolism remain poorly understood.

The retromer complex, composed of vacuolar protein sorting 35 (VPS35), VPS26, and VPS29, is a conserved endosomal sorting complex that mediates retrograde trafficking of transmembrane cargos from endosomes to the trans-Golgi network or plasma membrane [28–30]. VPS35 functions as the core cargo recognition subunit and is essential for proper recycling of multiple transmembrane proteins, including metabolic transporters, nutrient receptors, and signaling molecules [31–33]. Dysregulation of VPS35 has been implicated in multiple neurodegenerative diseases, including PD, AD, ADRD, and ALS, where its loss disrupts endosomal transport, mitochondrial dynamics, and cellular metabolism [33–43].

Notably, the D620N missense mutation in VPS35 causes autosomal-dominant familial PD, underscoring its causal role in human PD pathology [34, 36, 43]. Reduced VPS35 expression has been reported in the hippocampus of AD patients, frontal lobe of FTD patients, and motor neurons of ALS [35, 37, 39, 42], suggesting a broader contribution of VPS35 dysfunction to neurodegeneration. VPS35 is widely expressed in the brain, including neurons, microglia, ependymal cells, and astrocytes [38, 42, 44–47]. While its functions in mouse neurons, microglia, and ependymal cells have been characterized [38, 40–42, 44], the role of VPS35 in astrocytes remains largely unexplored. Given the central function of astrocytes in angiogenesis, BBB formation, and metabolic homeostasis, defining astrocytic VPS35’s role in BV and astrocyte development may uncover novel mechanisms underlying astrocyte regulation of BV development and neurodegenerative disease.

Here, we investigated the role of astrocytic VPS35 in postnatal vascular and astrocyte development. Astrocyte-specific deletion of VPS35 markedly reduced astrocyte density as well as vascular density, branching, endothelial proliferation, and activity-dependent angiogenesis. Mechanistically, loss of VPS35 in astrocytes led to intracellular iron accumulation, likely due to reduced iron exporter ferroportin (FPN), resulting in decreased HIF1α stability and VEGFa expression. In parallel, iron accumulation in Vps35-deficient astrocytes triggered ferroptosis, impairing astrocyte maturation and reducing astrocyte survival. Together, these findings establish VPS35 as a key regulator of astrocytic iron metabolism to promote angiogenesis and to prevent astrocyte ferroptosis during postnatal brain development.

## Results

### Necessity of astrocytic Vps35 for neonatal vascular development and adult vascular maintenance

To investigate the role of astrocytic Vps35 in cerebral BV development, we generated an inducible astrocyte-specific Vps35 knockout mouse line (Vps35^GfapcreER^) by crossing mice carrying floxed Vps35 alleles (Vps35^f/f^) with GFAP-CreER mice (Fig. S1A). Both GFAP-CreER and Vps35^GfapcreER^ mice were further crossed with the Ai3 Cre reporter line, which expresses GFP upon Cre-mediated recombination, to generate GFAP-CreER;Ai3 control mice and Vps35^GfapcreER^; Ai3 mutant mice, respectively.

Because angiogenesis in the mouse brain predominantly occurs during the first three postnatal weeks and culminates in vascular maturation around postnatal day 21 (P21) [48–50], mice were treated with tamoxifen (TAM; 75 mg/kg/day, once daily for three consecutive days) from P7 to P9 and sacrificed at P21 (Fig. S1A). Western blot of P21 S1 cortex indicated that VPS35 protein levels were decreased (Fig. S1B, C). This reduction was accompanied by lower levels of VPS26A and VPS29, other essential components of the retromer complex (Fig. S1B, C), consistent with impaired retromer complex integrity [28, 51]. Further RNAscope analysis revealed abundant Vps35 mRNA expression in Glast1^+^ astrocytes in control mice, whereas Vps35 mRNA was depleted in Glast1^+^ astrocytes, but not in Glast1^−^ cells, in the neocortex of Vps35^GfapcreER^ mice (Fig. S1D, E), demonstrating a specific and efficient depletion of Vps35 in mutant astrocytes. Notably, overall body weight and body size of mutant mice were comparable to those of control littermates (Fig. S1F).

**Fig. 1:**
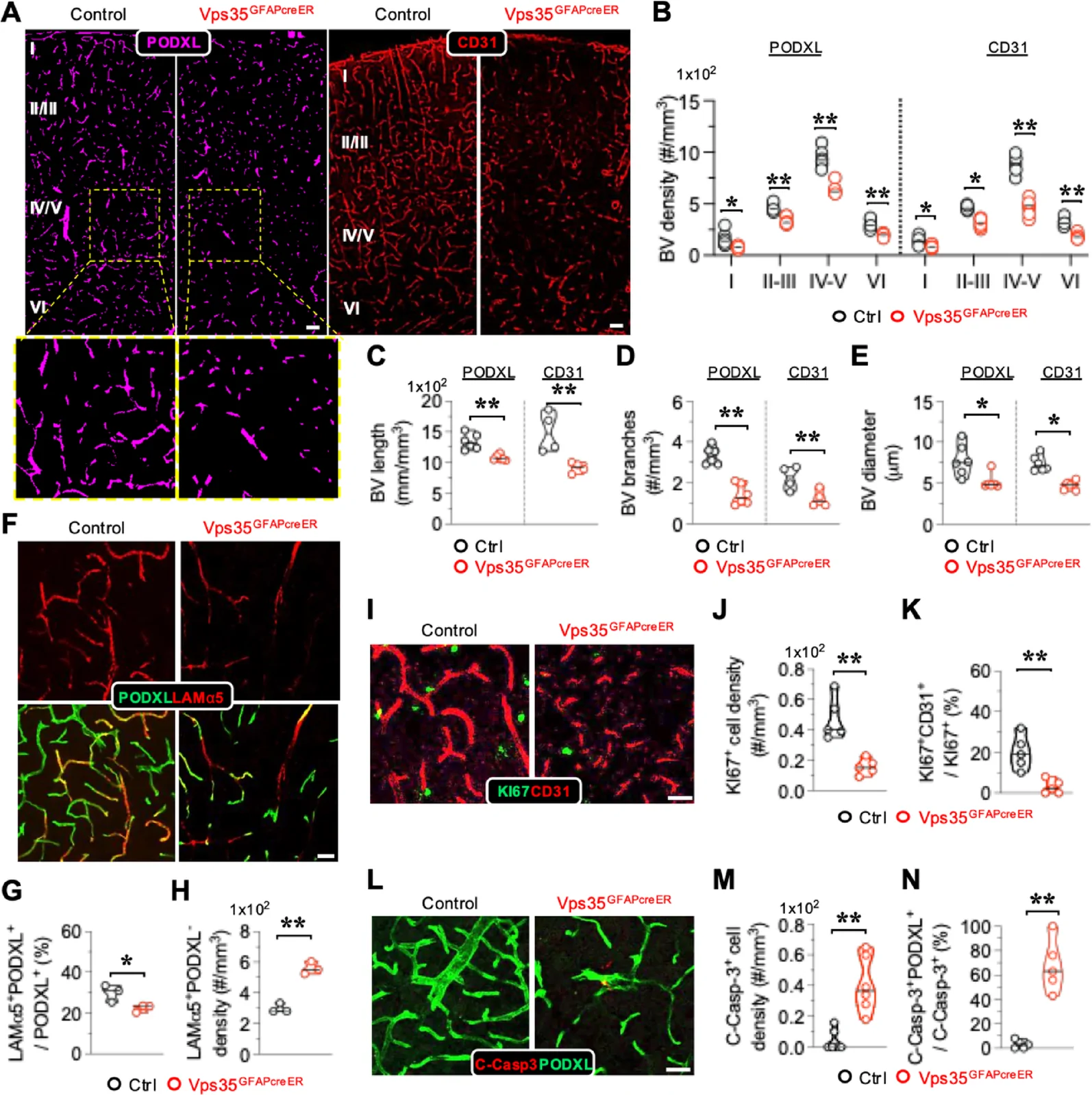
Requirement of astrocyte Vps35 for neonatal vascular development. **A** Representative immunostaining images of blood vessels (BVs) across cortical layers I–VI of the S1 cortex labeled with PODXL⁺ (magenta) and CD31⁺ (red) in control and Vps35^GFAPcreER^ mice. Higher-magnification images highlight PODXL⁺ BVs in layers IV–V. **B** Quantification of PODXL⁺ and CD31⁺ BV density (#/mm^3^) across cortical layers. **C**–**E** Quantification of PODXL⁺ and CD31⁺ BV morphology, including vessel length (mm/mm^3^; C), branch number (#/mm^3^; D), and vessel diameter (µm; **E**). **F** Representative immunostaining images of PODXL⁺ BVs (green) and Laminin-α5⁺ (red), a basement membrane marker associated with vessel maturation and stability. **G** Quantification of the percentage of PODXL⁺ BVs associated with Laminin-α5. **H** Quantification of Laminin-α5⁺ PODXL⁻ cell density (#/mm^3^). **I** Representative immunostaining images of Ki67 (green) and CD31 (red) to assess endothelial proliferation. **J** Quantification of Ki67⁺ cell density (#/mm^3^). **K** Quantification of the percentage of Ki67⁺CD31⁺ cells among total Ki67⁺ cells. **L** Representative immunostaining images of PODXL (green) and cleaved caspase-3 (cCasp3; red) to assess endothelial apoptosis. **M** Quantification of cCasp3⁺ cell density (#/mm^3^). **N** Quantification of the percentage of cCasp3⁺PODXL⁺ cells among total cCasp3⁺ cells. Data are presented as mean ± SD; n = 6 mice per group unless otherwise indicated; unpaired Student’s t-test (*p < 0.05, **p < 0.01). Scale bars: 50 µm (**A**, **F**); 25 µm (**I**, **L**)

We next examined cortical BVs at P21 in tamoxifen-treated Vps35^GFAPcreER^ mice and their control littermates (GFAP-CreER or Vps35^f/f^)(TAM administered at P7–P9). BVs were visualized by immunostaining with antibodies against PODXL and CD31, established markers of BV-ECs (Fig. 1A). Compared with controls, Vps35^GFAPcreER^ mice exhibited a marked reduction in BV density across all cortical layers (Fig. 1B). Quantitative analyses further revealed significant decreases in BV length, branching complexity, and vessel diameter in mutant mice (Fig. 1C, D, E). These findings suggest that astrocytic Vps35 is required for proper vascular formation and maturation during this critical postnatal developmental period.

To determine whether additional vascular components were affected, we examined the vascular basement membrane by laminin-α5 (LAMα5) immunostaining in combination with PODXL to assess vessel coverage. Quantitative analysis revealed a significant reduction in the proportion of LAMA5^+^PODXL^+^ over total PODXL^+^ BVs in Vps35^GFAPcreER^ mice compared with controls (Fig. 1F–G). Notably, in mutant mice we frequently observed regions with robust LAMα5 staining but little or no PODXL signal (Fig. 1F, H), implicating that basement membrane structures can persist following EC loss. These findings indicate that ECs are the primary vascular component affected by astrocytic Vps35 deficiency.

Given the pronounced reduction in ECs in the mutants, we next investigated whether astrocytic Vps35 loss impacts EC proliferation and/or survival. Immunostaining for the proliferation marker Ki67 showed reduced overall Ki67^+^ cell density and a decreased proportion of Ki67^+^CD31^+^ over total vessels in mutant mice (Fig. 1I–K). In contrast, assessment of the apoptotic marker cleaved caspase-3 revealed a significant increase in caspase-3^+^ cell density, as well as an elevated proportion of caspase-3^+^PODXL^+^ cells, in Vps35^GFAPcreER^ mice relative to controls (Fig. 1L–N), indicating enhanced endothelial cell death. Together, these results suggest that the vascular deficits observed in Vps35^GFAPcreER^ mice arise from both reduced proliferative capacity and EC survival.

To determine whether the vascular phenotype observed in the cerebral cortex extended to other brain regions, we performed vascular analyses in the hippocampus of Vps35^GFAPcreER^ and control mice. Immunostaining for PODXL revealed that vessel density and diameter complexity was significantly different between Vps35^GFAPcreER^ and control mice in the hippocampus (Fig. S2A–C). However, blood vessel branching was not significantly reduced in Vps35^GFAPcreER^ mice compared to controls (Fig. S2D), indicating that astrocytic VPS35 loss affects vascular morphology in the hippocampus . Together, these data suggest that the vascular consequences of astrocytic VPS35 deletion are not entirely restricted to the cerebral cortex, but that the nature and severity of the phenotype differs across brain regions.

We further asked whether astrocytic Vps35 is required for BV homeostasis in adult mice. To address this, 1-month-old (MO) Vps35^GFAP−CreER^ mice and their control littermates (GFAP-CreER or Vps35^f/f^) were treated with TAM for three consecutive days and sacrificed at 2-MO (Fig. S3A). Cortical sections were then immunostained for the endothelial marker CD31 (Fig. S3B). Quantitative analyses revealed significant reductions in BV denisty, length, branching complexity, and vessel diameter in Vps35^GFAPcreER^ mice compared with controls (Fig. S3C–F). Together, these results indicate that astrocytic Vps35 is required for not only the formation of cerebral BV during neonatal development, but also their maintenance in young adult age.

### Requirement of astrocytic Vps35 for activity-induced angiogenesis and blood flow

**Fig. 2:**
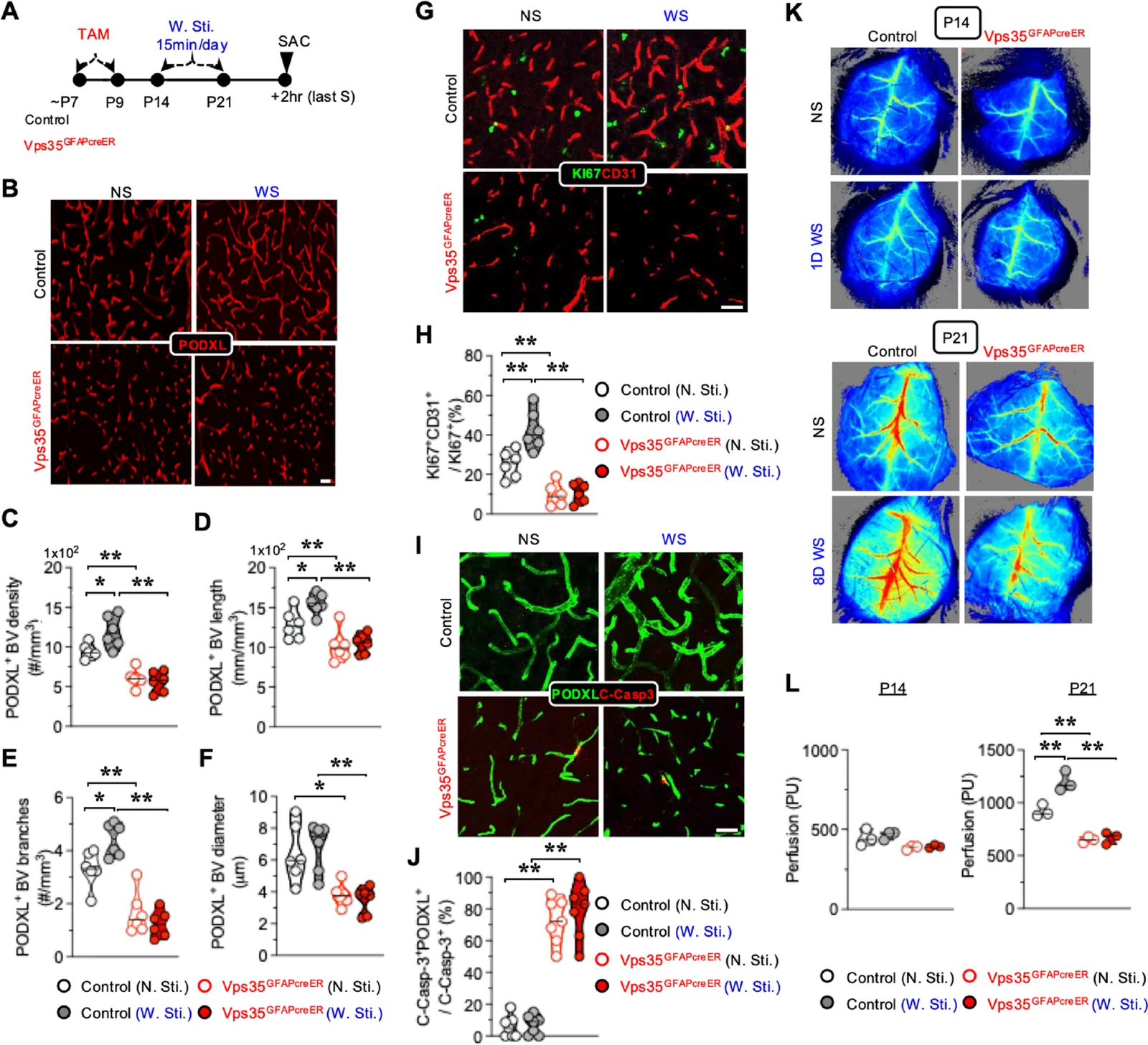
Necessity of astrocytic Vps35 for activity-dependent angiogenesis. **A** Schematic of the experimental design. Vps35^f/f^ and Vps35^GFAPcreER^ mice underwent unilateral whisker stimulation (WS) for 15 min/day from postnatal day (P)14 to P21; the contralateral non-stimulated (NS) side served as an internal control. **B** Representative immunostaining images of PODXL⁺ BVs across cortical layers IV–VI of the S1 cortex with or without WS. **C**–**F** Quantification of PODXL⁺ BV density (**C**), vessel length (**D**), branch number (**E**), and vessel diameter (**F**). **G** Representative immunostaining images of Ki67 (green) and CD31 (red). **H** Quantification of the percentage of Ki67⁺CD31⁺ cells among total Ki67⁺ cells. **I** Representative immunostaining images of PODXL (green) and cCasp3 (red). **J** Quantification of the percentage of cCasp3⁺PODXL⁺ cells among total cCasp3⁺ cells. **K** Representative bilateral cerebral blood-flow maps acquired by laser-speckle contrast imaging (LSCI) at P14 and P21 with or without WS. **L** Quantification of perfusion units (PU) at P14 and P21.Data are presented as mean ± SD; n = 6 mice per group for histological analyses and n = 3 mice per group for LSCI; one-way ANOVA (*p < 0.05, **p < 0.01). Scale bar: 25 µm

Neuronal activity is known to promote angiogenesis during neonatal brain development [27, 50, 52]. To determine whether neuronal activity–induced BV formation depends on astrocytic VPS35, control and Vps35^GFAPcreER^ mice, treated with tamoxifen at P7–P9, were subjected to daily whisker stimulation (WS) beginning at P14 for eight consecutive days (Fig. 2A). In control mice, BV density was significantly increased in layers IV–V of the contralateral (whisker stimulated) barrel cortex compared with the ipsilateral non-stimulated (NS) side, in line with previous reports [27, 52]. In contrast, Vps35^GFAPcreER^ mice failed to exhibit this activity-dependent vascular response (Fig. 2B–F), suggesting the necessity of astrocytic VPS35 in this event.

To further characterize this phenotype, we examined whether WS could promote BV formation and/or prevent EC loss in mutant mice by assessing EC proliferation and apoptosis. As shown in Fig. 2G-H, WS increased Ki67^+^CD31^+^ cells in control S1 cortex, indicating an increase in EC proliferation. However, no significant differences were observed between stimulated and non-stimulated S1 cortex in Vps35^GFAPcreER^ mice for either Ki67 marked proliferation or active Caspase 3 labeled cell death (Fig. 2G–J).

In parallel, BV function was evaluated by measuring cerebral blood flow using laser speckle contrast imaging at P14 (the first day of WS) and P21 (8 days of WS). Although no differences were detected between control and mutant mice at P14, chronic WS significantly increased cerebral blood flow in control mice by P21 (Fig. 2K–L). In contrast, Vps35^GFAPcreER^ mice failed to show this increase and instead exhibited significantly reduced blood flow compared with controls, regardless of stimulation (Fig. 2K–L).

Together, consistent with previous studies [27, 52], these results demonstrate that WS promotes the formation and maturation of cortical vascular networks in control mice, and establish that astrocytic VPS35 is required for basal and neuronal activity–driven angiogenesis during postnatal cortical development.

### Reduced meningeal BV density and diameter in Vps35^GFAP−CreER^ mice

Given that laser speckle contrast imaging primarily captures cortical surface and meningeal blood flow, the observed reduction in perfusion prompted us to investigate whether meningeal vasculature is also affected by loss of astrocytic Vps35. To address this question, we employed a thin transverse sectioning approach to isolate the superficial cortical layer (1-mm depth) for whole-mount co-immunostaining of the pia mater [53] (Fig. S4A). The pia mater, the innermost meningeal layer, contains large arteries that supply blood to the brain as well as large veins involved in metabolic waste clearance [49, 54]. Consistent with the pronounced cerebrovascular phenotype observed in the cortex, immunostaining of the meninges revealed a significant reduction in PODXL⁺ endothelial density accompanied by increased IBA1⁺ microglial signal in Vps35^GFAPcreER^ mice (Fig. S4B, C). To further assess arterial integrity, we performed smooth muscle actin (SMA) staining and observed a trend toward reduced arterial vessel density that did not reach statistical significance, together with a significant decrease in SMA⁺ vessel width in mutant mice (Fig. S4D, E). Collectively, these findings indicate that astrocytic VPS35 deletion not only impairs cortical angiogenesis but also disrupts meningeal vascular development, likely contributing to the reduced cerebral blood flow observed in these animals.

### Impairments in HIF1α-VEGFa signaling pathway in Vps35-KO astrocytes

**Fig. 3:**
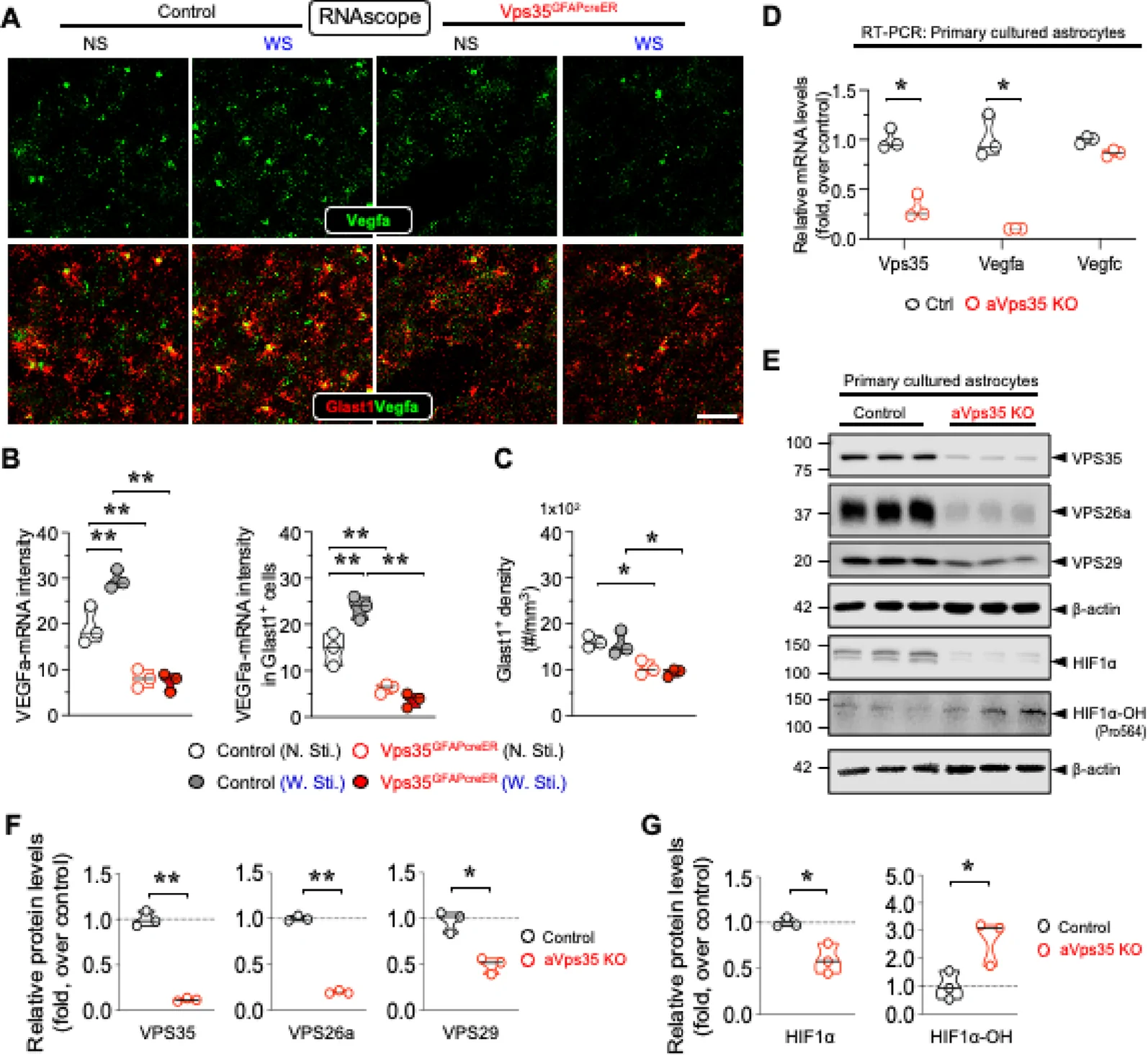
Impaired HIF1α–VEGFA signaling in Vps35-deficient astrocytes. **A** Representative RNAscope images showing Vegfa mRNA (green) and Glast1 (red) in layers IV/V of the S1 cortex of control and Vps35^GFAPcreER^ mice with or without whisker stimulation. **B** Quantification of Vegfa mRNA signal intensity and Vegfa⁺GLAST1⁺ cell density. **C** Quantification of GLAST1⁺ astrocyte density in the S1 cortex. **D** Quantitative RT-PCR analysis of Vegfa mRNA expression in primary astrocyte cultures derived from control (Vps35^f/f^ or GFAPcre) and Vps35^GFAPCre^ mice. **E** Representative immunoblots of retromer complex components and HIF1α signaling proteins in primary astrocyte lysates. **F** Quantification of retromer complex protein levels shown in (**E**). **G** Quantification of total HIF1α and hydroxylated HIF1α (HIF1α-OH) levels shown in (**E**). Data are presented as mean ± SD; n = 3 mice or independent cultures per group; unpaired Student’s t-test or one-way ANOVA as indicated (*p < 0.05, **p < 0.01). Scale bar: 50 µm

To investigate the molecular mechanisms underlying impaired basal and activity-dependent angiogenesis in astrocytic Vps35 mutants, we focused on the HIF1α–VEGFa signaling pathway, because it is highly active and essential for angiogenesis during this postnatal developmental window [49, 52, 55]. Using RNAscope, we assessed Vegfa mRNA expression together with the astrocytic marker Glast1 in the S1 cortex, with particular emphasis on layer IV, in control and Vps35^GFAPcreER^ mice with or without WS (Fig. 3A). In control mice, WS significantly increased Vegfa mRNA levels (Fig. 3A, B), consistent with previous reports [52, 56]. In contrast, Vegfa mRNA expression was markedly reduced in mutant mice, and WS failed to restore Vegfa expression to control levels (Fig. 3A, B). Quantitative analysis revealed a significant reduction in the intensity of Vegfa in Glast1⁺ cells in Vps35^GFAPcreER^ mice (Fig. 3A, B). While WS significantly increased the intensity of Vegfa in Glast1⁺ cells in control mice, this activity-dependent response was blunted in mutants and could not be rescued by stimulation (Fig. 3B). Notably, we observed a significant decrease in Glast1⁺ astrocyte density in the Vps35^GFAPcreER^ cortex (Fig. 3C), implicating a deficit in astrocyte development. Together, these findings demonstrate a robust reduction in astrocytic Vegfa expression in the Vps35^GFAPcreER^ cortex under both basal and stimulated conditions, supporting the conclusion that diminished Vegfa signaling contributes to the angiogenic deficits observed in mutant mice.

To further determine whether these effects are cell autonomous, we examined Vegfa expression in primary astrocyte cultures derived from P2 pups carrying a floxed Vps35 allele (Vps35^f/f)^ crossed with GFAP-Cre. RT–qPCR confirmed efficient depletion of Vps35 in cultured mutant astrocytes and revealed a concomitant reduction in Vegfa mRNA levels, whereas expression of Vegfc, another VEGF family member, remained unchanged (Fig. 3D). Western blot analysis demonstrated markedly reductions in VPS35, VPS6a, and VPS29 protein levels in mutant cultured astrocytes (Fig. 3E, F), demonstrating again the efficiency of VPS35 depletion, and consistent with the requirement of VPS35 for retromer complex assembly and/or stability [28, 51]. Importantly, western blot examining HIF1α, an essential transcription factor for VEGFa expression [57, 58], in culture astrocytes revealed reduced HIF1α protein levels accompanied by an increase in hydroxylated HIF1α (HIF1α-OH) in the mutant cells (Fig. 3E, G), indicating impaired HIF1α stabilization. Together, these findings suggest that astrocytic VPS35 is necessary to promote VEGFa expression, likely by limiting HIF1α hydroxylation to stabilize HIF1α.

### Increased intracellular irons in Vps35-KO astrocytes in culture and in neocortex

**Fig. 4:**
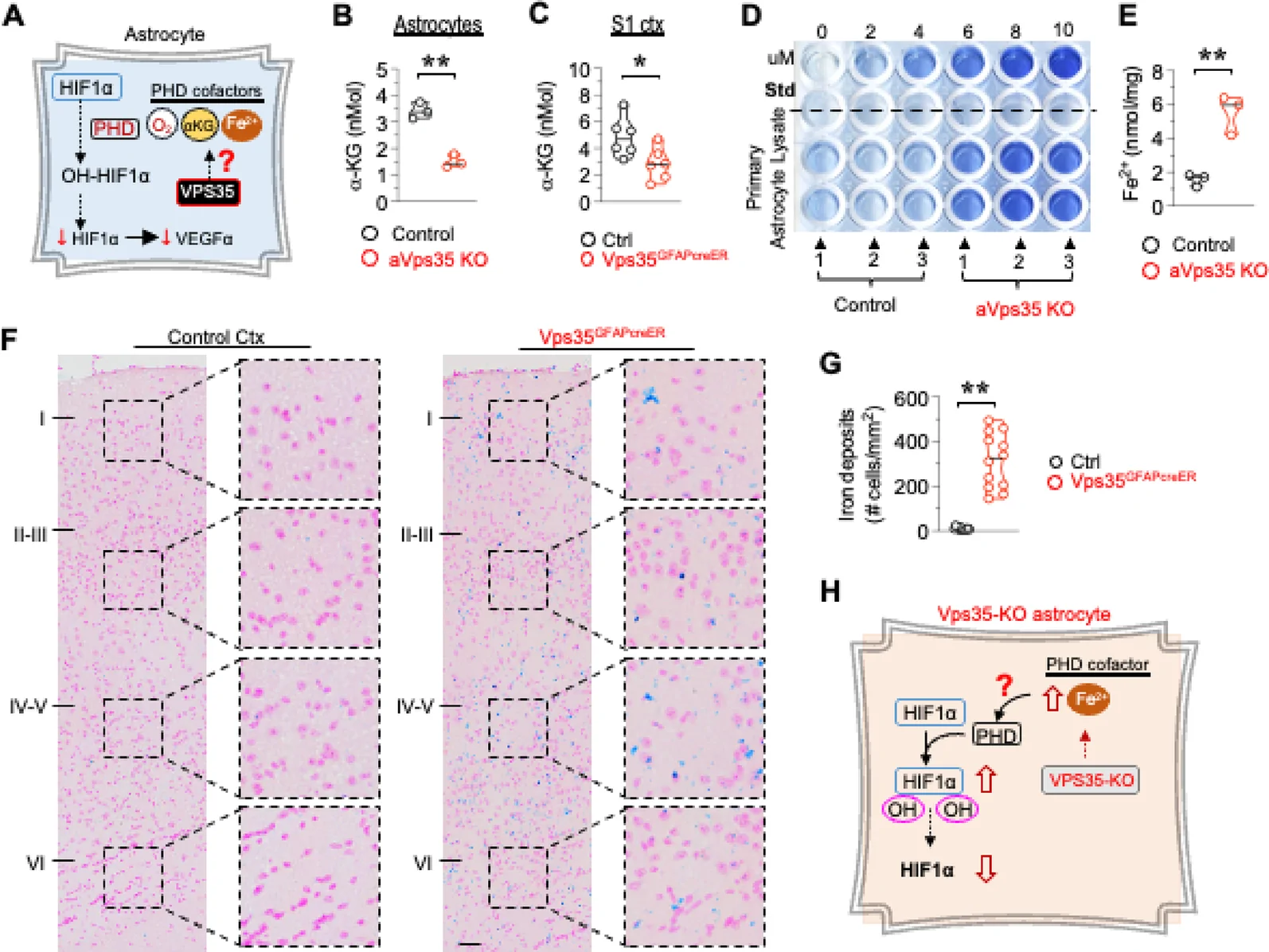
Increased iron levels, but decreased α-KG, in Vps35-KO astrocytes in culture and in vivo. **A** Schematic illustrating HIF1α degradation mediated by prolyl hydroxylase domain (PHD) enzymes, which require oxygen, α-ketoglutarate (α-KG), and ferrous iron (Fe^2^⁺) as cofactors. **B** Measurement of α-KG levels in primary cultured astrocytes derived from control and Vps35^GFAPcre^ mice. **C** Measurement of α-KG levels in S1 cortical tissue lysates from control and Vps35^GFAPcreER^ mice (P21). **D** Representative colorimetric iron assay measuring intracellular Fe^2^⁺ levels in primary astrocytes. **E** Quantification of intracellular Fe^2^⁺ levels. **F** Representative Prussian blue staining showing ferric iron (Fe^3^⁺) deposition in the cortex (P21). **G** Quantification of Fe^3^⁺ deposit density. **H** Model illustrating iron-dependent HIF1α destabilization following astrocytic Vps35 loss. Data are presented as mean ± SD; n = 3 mice or independent cultures per group; unpaired Student’s t-test (*p < 0.05, **p < 0.01). Scale bar: 50 µm

HIF1α hydroxylation, a critical step that targets HIF1α for proteasomal degradation, is mediated by prolyl hydroxylase domain (PHD) enzymes, which require oxygen, α-ketoglutarate (α-KG), and ferrous iron as essential cofactors [59, 60](Fig. 4A). To investigate how astrocytic VPS35/retromer regulates HIF1α stability, we examined these PHD cofactors in control and Vps35-deficient astrocytes. We first measured α-KG levels in cultured astrocytes from control and mutant mice and observed a dramatic decrease in mutant astrocytes (Fig. 4B). To verify this, we further quantified α-KG levels in somatosensory cortex tissue from control and Vps35^GFAPcreER^ mice and also detected a significant reduction in α-KG in mutant samples (Fig. 4C). Because reduced α-KG would be predicted to diminish PHD activity and stabilize HIF1α, this reduction is unlikely to explain the lower HIF1α levels observed in Vps35-deficient astrocytes.

We next examined iron availability in cultured astrocytes, another critical cofactor for PHD activity [59](Fig. 4A). Using a ferrous iron assay, we detected a marked increase in intracellular iron levels in mutant astrocytes compared with controls (Fig. 4D–E). To validate these findings in vivo, we performed Prussian blue staining on cortical sections from P21 control and Vps35^GFAPcreER^ mice, which revealed a pronounced increase in iron-positive cells, likely glial cells, in the mutant cortex (Fig. 4F–G). Together, these results suggest that astrocytic Vps35 deficiency results in intracellular iron overload, leading to a speculation that iron accumulation may play a role in enhancing PHD-dependent HIF1α hydroxylation and thereby reduces HIF1α stability in Vps35-deficient astrocytes (Fig. 4H).

### Restoration of HIF1α-VEGFa signaling and BV density in Vps35-KO astrocytes via iron chelation

**Fig. 5:**
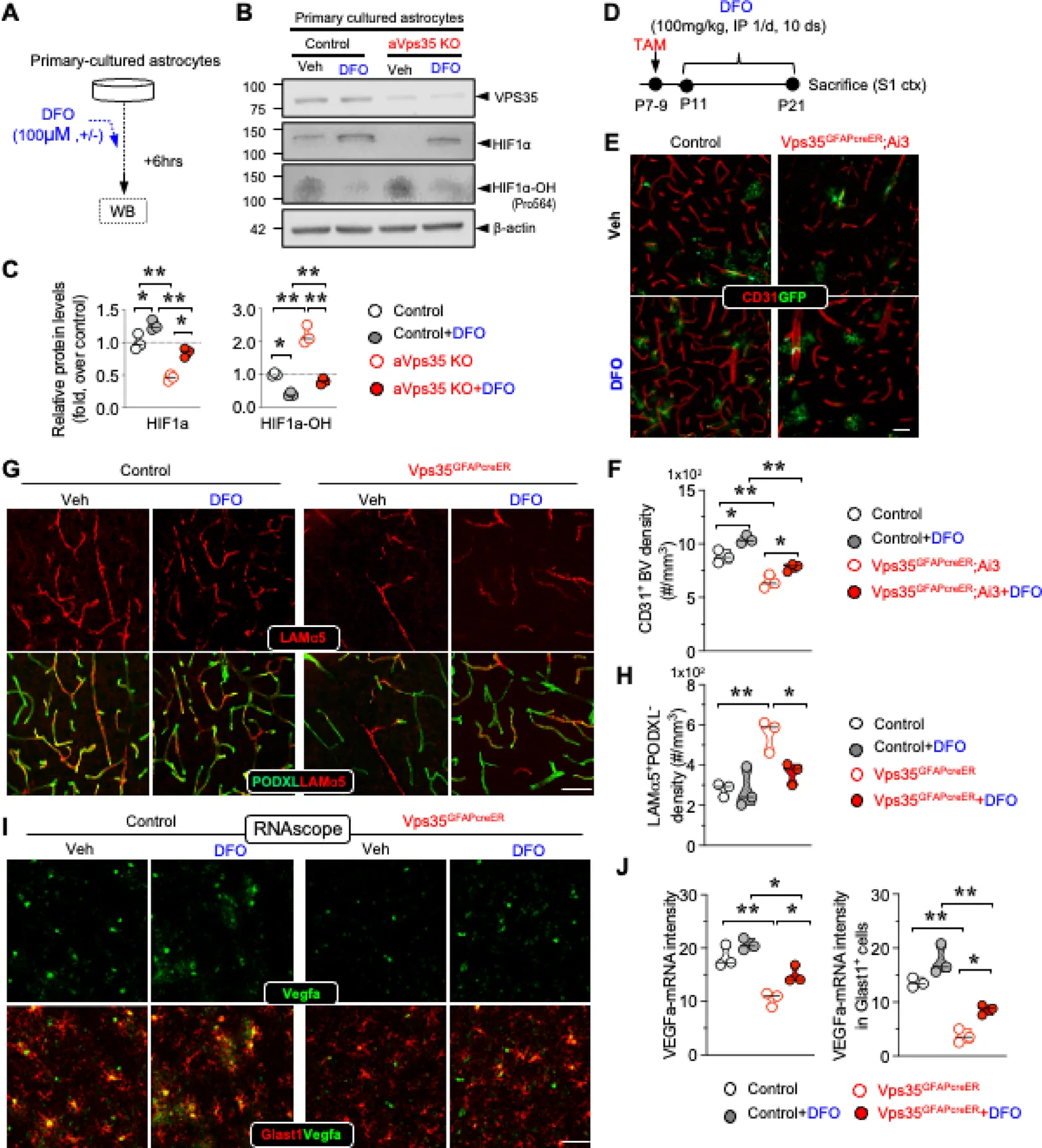
Restoration of HIF1α–VEGFA signaling and vascular density in Vps35-deficient astrocytes. **A** Schematic of iron chelation using deferoxamine (DFO; 100 µM, 6 h) in primary astrocyte cultures derived from control and Vps35^GFAPcre^ mice. **B** Representative immunoblots of HIF1α and hydroxylated HIF1α in control and Vps35-deficient astrocytes. **C** Quantification of HIF1α and hydroxylated HIF1α levels shown in (**B**). **D** Schematic of in vivo iron chelation using DFO (100 mg/kg) administrated into P11–P21 control (GFAPcreER;Ai3) and Vps35^GFAPcreER^;Ai3 mice (i.p. once daily for 10 days). **E** Representative immunostaining of CD31 and GFP in control and mutant mice with or without DFO treatment. **F** Quantification of CD31⁺ BV density (#/mm^3^). **G** Representative immunostaining of PODXL and Laminin-α5. **H** Quantification of Laminin-α5⁺ PODXL⁻ cell density (#/mm^3^). **I** Representative RNAscope images showing Vegfa and Glast1 mRNA expression. **J** Quantification of Vegfa mRNA signal intensity and Vegfa⁺GLAST1⁺ cell density. Data are presented as mean ± SD; n = 3 mice or independent cultures per group; one-way ANOVA (*p < 0.05, **p < 0.01). Scale bar: 50 µm

To test the above speculation, we asked whether iron chelation could rescue HIF1α signaling and associated vascular deficits. Primary cultured astrocytes were treated with the iron chelator deferoxamine (DFO; 100 µM) for 6 h (Fig. 5A), and cell lysates were subjected to western blot analysis. As shown in Fig. 5B, DFO treatment restored HIF1α protein levels in Vps35-deficient astrocytes. Consistent with this effect, levels of hydroxylated HIF1α (HIF1α-OH) were significantly reduced in DFO-treated mutant cells (Fig. 5C), supporting the view that iron chelation attenuates Fe^2+^-dependent PHD activity and thereby limits HIF1α hydroxylation and degradation.

We then assessed whether iron chelation could rescue vascular phenotypes in vivo. Control and Vps35^GFAPcreER^;Ai3 mice were treated with tamoxifen at P7–P9, followed by daily intraperitoneal injections of DFO (100 mg/kg) from P11 to P21 (Fig. 5D). Cortical brain sections were subsequently co-immunostained for CD31 (the endothelial marker) and GFP (the GFAP-CreER^+^ astrocytes). As shown in Fig. 5E, DFO treatment increased CD31^+^ BV density in control mice compared with untreated controls. Importantly, CD31^+^ BV density was also significantly elevated in DFO-treated Vps35^GFAPcreER^ mice relative to untreated mutants (Fig. 5F), demonstrating a partial rescue of the vascular deficit.

We further evaluated vascular basement membrane integrity by immunostaining for LAMα5. Consistent with our earlier observations of increased LAMα5⁺PODXL⁻ vessels in mutant mice, DFO treatment significantly reduced the density of LAMα5⁺PODXL⁻ vessels in Vps35^GFAPcreER^ mice (Fig. 5G, H), further supporting a partial normalization of vascular structure following iron chelation.

Finally, we tested whether iron chelation could increase Vegfa mRNA levels using RNAscope. Indeed, upon DFO treatment, Vegfa-mRNAs in Glast1^+^ astrocytes of Vps35 mutant mice were elevated (Fig. 5I, J). Together, these results suggest that iron accumulation in Vps35 deficient astrocytes contributes to the impaired HIF1α-VEGFa signaling and angiogenesis (Fig. 5I, J).

### Ferroptosis in Vps35-KO astrocytes that contributes to the astrocyte-loss

**Fig. 6:**
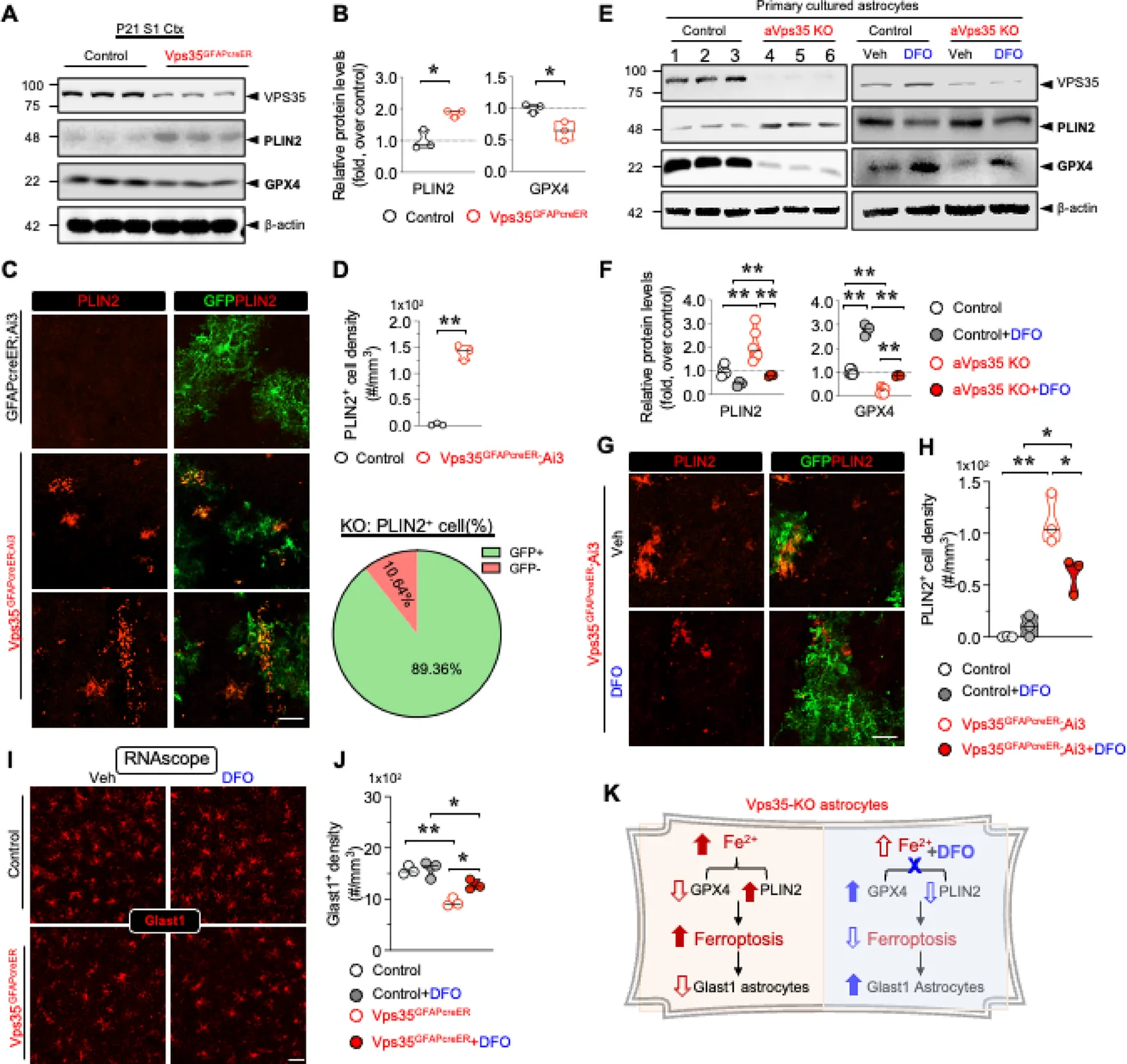
Ferroptotic cell death in Vps35-deficient astrocytes that is partially rescued by iron chelation. **A** Representative immunoblots of ferroptosis-related proteins PLIN2 and GPX4 in S1 cortical tissue lysates from control and Vps35^GFAPcreER^ mice. **B** Quantification of PLIN2 and GPX4 protein levels shown in (**A**). **C** Representative immunostaining of PLIN2 (red) and GFP (green) in GFAPcreER;Ai3 and Vps35^GFAPcreER^;Ai3 mice. **D** Quantification of PLIN2⁺ cell density and percentage of PLIN2⁺GFP⁺ astrocytes. **E** Representative immunoblots of PLIN2 and GPX4 in primary astrocyte cultures derived from control and Vps35^GFAPcre^ mice with or without DFO treatment. **F** Quantification of PLIN2 and GPX4 protein levels shown in (**E**). **G** Representative immunostaining of PLIN2 and GFP in vivo following DFO treatment in Vps35^GFAPcreER^;Ai3 mice. **H** Quantification of PLIN2⁺ cell density. **I** Representative RNAscope images showing Glast1 expression from control and Vps35^GFAPcreER^ mice. **J** Quantification of Glast1 mRNA density. **K** Model illustrating ferroptotic vulnerability induced by astrocytic Vps35 loss and partial rescue by iron chelation. Data are presented as mean ± SD; n = 3 mice or independent cultures per group; unpaired Student’s t-test or one-way ANOVA (*p < 0.05, **p < 0.01). Scale bars: 20 µm (**C**, **G**); 40 µm (**I**)

Given the increase of intracellular irons in mutant astrocytes (Fig. 4) and the reduction in astrocyte density observed in Vps35^GFAPcreER^ mice, as indicated by decreased Glast1^+^ cells in our RNAscope analysis (Fig. 3C), we investigated whether ferroptosis, a regulated form of cell death driven by iron overload and lipid peroxidation [61, 62], occurred in Vps35 mutant astrocytes and contributed to the astrocyte loss. To assess this, we examined the expression of established ferroptosis markers, including perilipin 2 (PLIN2), a lipid droplet associated protein that accumulates during lipid peroxidation, and glutathione peroxidase 4 (GPX4), a key antioxidant enzyme that suppresses ferroptosis by reducing lipid hydroperoxides [63–65]. Remarkably, western blot analysis of S1 cortex from P21 control and Vps35^GFAPcreER^ mice revealed significantly increased PLIN2 levels and decreased GPX4 levels in mutants (Fig. 6A, B), supporting the view for enhanced ferroptotic signaling in Vps35^GFAPcreER^ S1 cortex.

To determine the cell type and validate the occurrence of ferroptosis in vivo, we performed PLIN2 co-immunostaining with GFP in Vps35^GFAPcreER^;Ai3 mice (Fig. 6C). Quantitative analysis revealed that the majority of PLIN2^+^ cells were associated with GFP^+^ astrocytes, with 89.36% co-localizing with GFP and 10.64% localized to GFP negative cells (Fig. 6D).

To further verify ferroptosis occurred in mutant astrocytes, we examined the levels of the ferroptosis associated markers (PLIN2 and GPX4) in primary cultured astrocytes by western blot. Again, increased PLIN2 and decreased GPX4 were detected in VPS35-KO astrocytes (Fig. 6E, F), indicating that this phenotype is astrocyte selective and cell autonomous. Notably, treatment of mutant astrocytes with the iron chelator DFO significantly reduced PLIN2 levels while partially restoring GPX4 expression (Fig. 6E, F). Importantly, DFO treatment in vivo reduced PLIN2^+^ cell density (Fig. 6G, H) and increased Glast1^+^ astrocyte density (Fig. 6I, J) in mutant mice. Together, these results support the view that iron overload induced ferroptosis contributes to the astrocyte loss in the absence of Vps35 (Fig. 6K).

### Altered astrocyte morphology and impaired astrocyte maturation in ***Vps35***^GFAP−CreER^ cortex

Given that the iron overload induced ferroptosis contributed to the astrocyte loss in Vps35^GFAPcreER^ mice, we further asked addressed whether astrocytic Vps35 regulates astrocyte morphogenesis and differentiation/maturation during this critical period. To this end, we used GFAP^creER^;Ai3 and Vps35^GFAPcreER^;Ai3 mice, which genetically label astrocytes with GFP, and performed high-resolution imaging of individual GFP^+^ astrocytes (Fig. S5A). Morphometric analysis using Imaris, revealed a significant reduction in soma area and total branch length in Vps35 deficient astrocytes (Fig. S5B), indicating impaired morphological maturation and reduced structural complexity.

To further assess whether astrocyte development or identity was altered, we examined the expression of multiple glial lineage and maturation markers (Fig. S6A, C, E). S100β is commonly expressed in astrocytes and is associated with calcium signaling and cytoskeletal regulation [66]. While overall S100β^+^ cell density was not significantly different between genotypes, it trended higher in mutant mice. Notably, the percentage of S100β^+^ cells co-labeled with GFP was significantly increased in mutants (Fig. S6B), suggesting an altered astrocytic maturation in the absence of Vps35.

We next examined Olig2, a transcription factor expressed in oligodendrocyte lineage and a subset of astrocyte progenitors during development [67], to determine whether astrocyte fate specification was altered. No significant differences were observed in Olig2^+^ cell density or the proportion of GFP^+^Olig2^+^ cells between control and mutant mice (Fig. S6D), indicating that the loss of Vps35 does not promote aberrant oligodendroglia lineage adoption by astrocytes.

Finally, we assessed glutamine synthetase (GS), a hallmark enzyme of mature astrocytes essential for glutamate-glutamine cycling and metabolic support of neurons [68, 69]. GS^+^ cell density and the percentage of GS^+^ cells co-labeled with GFP was significantly reduced in Vps35^GFAPcreER^;Ai3 mice (Fig. S6F). This finding suggests that astrocytic Vps35 deficiency impairs acquisition or maintenance of key metabolic features associated with astrocyte maturation.

### Decreased iron exporter FPN protein levels in Vps35-KO astrocytes

**Fig. 7:**
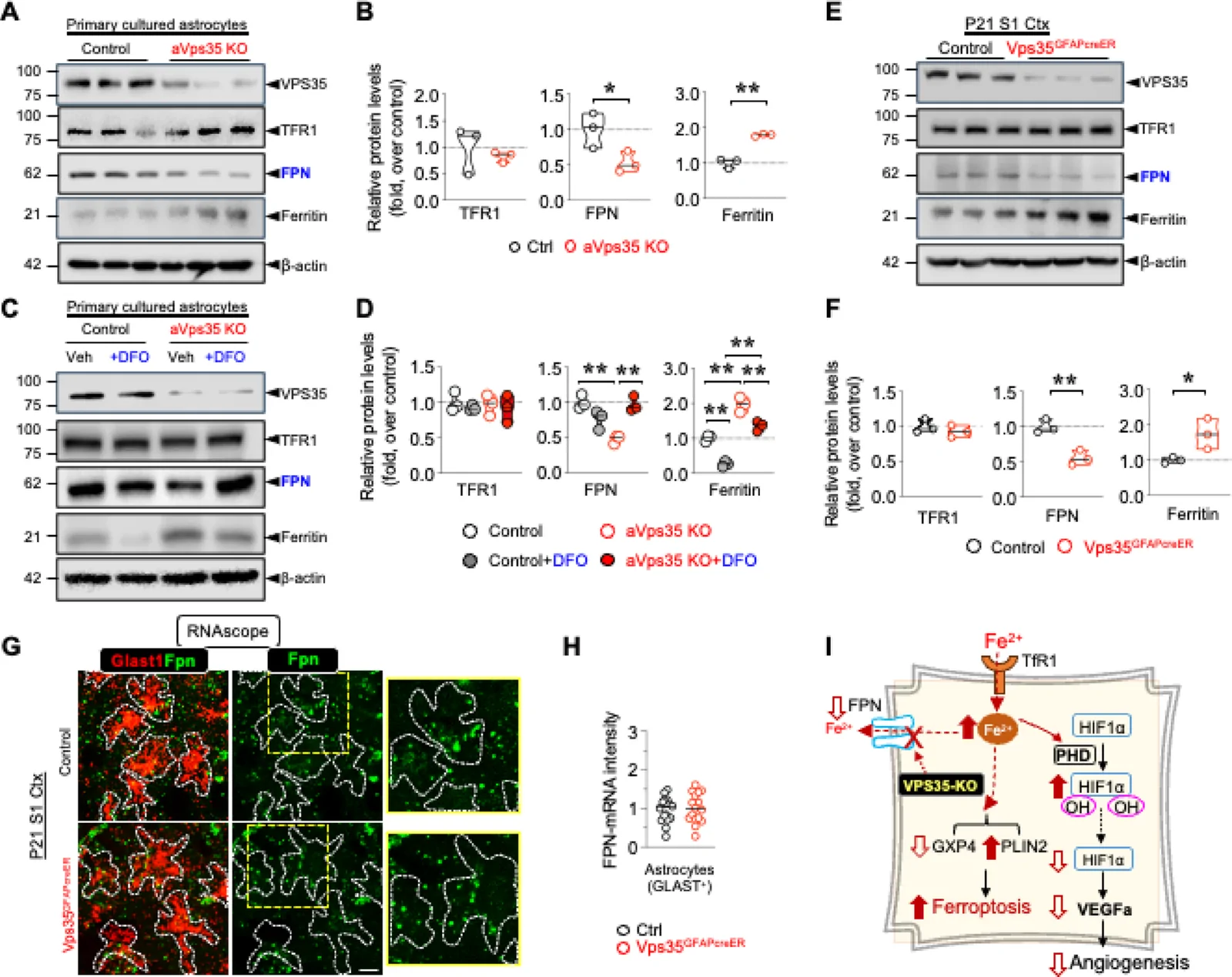
Decreased iron exporter FPN protein, but not mRNAs, in Vps35-KO astrocytes. **A** Representative immunoblots of transferrin receptor 1 (TFR1), ferroportin (FPN), and ferritin in primary astrocyte cultures derived from control and Vps35^GFAPcre^ mice. **B** Quantification of protein levels shown in (**A**). **C** Representative immunoblots of TFR1, FPN, and ferritin following DFO treatment. **D** Quantification of protein levels shown in (**C**). **E** Representative immunoblots of TFR1, FPN, and ferritin in cortical tissue lysates from control and Vps35^GFAPcreER^ mice. **F** Quantification of protein levels shown in (**E**). **G** Representative RNAscope images showing Glast1 and Fpn mRNA expression in the S1 cortex from control and Vps35^GFAPcreER^ mice. **H** Quantification of Fpn mRNA intensity. **I** Model illustrating iron accumulation, HIF1α destabilization, impaired angiogenesis, and ferroptosis resulting from astrocytic Vps35 loss. Data are presented as mean ± SD; n = 3 mice or independent cultures per group; unpaired Student’s t-test or one-way ANOVA (*p < 0.05, **p < 0.01). Scale bar: 10 µm

To understand how astrocytic VPS35/retromer regulates iron homeostasis, we asked whether proteins involved in regulating iron homeostasis are altered in Vps35-deficient astrocytes. Cellular iron levels are tightly controlled by coordinated regulation of iron import and export pathways, primarily mediated by transferrin receptor 1 (TFR1), which facilitates iron uptake, and FPN, an iron exporter [70–74]. We examined these proteins in primary cultured astrocytes from control and astrocytic Vps35 KO mice. Interestingly, western blot revealed a significant reduction in FPN levels in mutant astrocytes, whereas TFR1 levels remained unchanged (Fig. 7A, B), suggesting an impaired iron export rather than increased iron import for the intracellular iron accumulation. Consistent with increased intracellular iron levels ferritin levels, an iron storage protein that increases in response to elevated cellular iron (Fe [2]^+^), were significantly increased in mutant astrocytes (Fig. 7A, B).

We then determined whether iron chelation or DFO treatment could modulate these changes. Interestingly, DFO treatment significantly increased FPN levels in the mutant, but not control, astrocytes (Fig. 7C, D). This effect appeared to be specific for FPN, as TFR1 was unaffected by the DFO treatments (Fig. 7C, D). DFO treatment also reduced ferritin levels in the control and mutant cells (Fig. 7C, D). These results further indicate that iron chelation selectively restores iron export capacity without affecting iron uptake mechanisms.

We further verified the changes of these iron homeostasis regulators in vivo by western blot analysis. In agreement with the results from astrocyte cultures, Vps35 mutant cortex showed a reduction in FPN, an increase in ferritin, but no change in TFR1 levels (Fig. 7E, F). To determine whether FPN loss occurred at the transcriptional or post-transcriptional level, we assessed FPN mRNA expression using RNAscope (Fig. 7G). Quantitative analysis revealed no significant difference in FPN mRNA levels between control and mutant astrocytes (Fig. 7H), suggesting a post-transcriptional regulation by VPS35 and implicating that FPN might be a cargo of VPS35/retromer in astrocytes. Together, these findings suggest a working model that astrocytic Vps35 deficiency disrupts iron homeostasis likely through impaired FPN mediated iron export, leading to intracellular iron accumulation, ferroptosis, and subsequent decrease of astrocytes and BVs (Fig. 7I).

## Discussion

In this study, we identify astrocytic Vps35, a key component of the retromer, as a central regulator of postnatal cerebrovascular and astrocyte development and maintenance by linking iron homeostasis to astrocyte survival and angiogenic signaling. Conditional loss of Vps35 in astrocytes resulted in impaired basal and activity-dependent angiogenesis, reduced blood flow, and widespread vascular abnormalities affecting both cortical and meningeal vessels. Mechanistically, Vps35 deficiency in astrocytes led to intracellular iron accumulation, destabilization of HIF1Α, suppression of VEGFa expression, and induction of ferroptosis. The iron accumulation is associated with impaired FPN mediated iron export. Together, these findings lead to a working model depicted in Fig. 7I, revealing an astrocyte-intrinsic pathway through which retromer promotes astrocyte and neurovascular development and function.

Notably, vascular abnormalities were not restricted to cortical parenchyma but were also observed in meningeal vessels at the surface of the brain. Meningeal arteries and veins are essential for supplying blood to the cortex and for metabolic waste clearance, and defects at this level can profoundly influence cortical perfusion. The presence of reduced vessel density, altered arterial width, and increased IBA1^+^ macrophages in the meninges suggests that astrocytic Vps35 regulates vascular organization across multiple compartments of the neurovascular unit. To further examine regional specificity, we analyzed hippocampal vasculature in Vps35 KO mice. While branching were not significantly altered, blood vessel density was diameter was significantly reduced compared to controls (Fig. S2), indicating that VPS35 loss in astrocytes influences vascular morphology beyond the cortex, though the nature of the phenotype differs by region. This likely reflects known heterogeneity in astrocyte identity and angiogenic function across brain regions [75], with cortical astrocytes exhibiting a more pronounced dependence on VPS35-mediated angiogenic signaling during the postnatal window. Together, these observations demonstrate that astrocytic Vps35 influences vascular organization at both the meningeal and cortical levels, underscoring its widespread contribution to cerebrovascular integrity.

Astrocytes play a critical role in coordinating neuronal activity with vascular remodeling during postnatal brain maturation. Sensory-driven neuronal activity promotes angiogenesis by inducing astrocytic secretion of angiogenic factors, including VEGFa, thereby coupling neural demand to vascular supply [27, 52]. Consistent with this framework, whisker stimulation robustly increased vessel density, branching, and cerebral blood flow in control mice. In contrast, astrocytic Vps35 mutants failed to exhibit these activity-dependent vascular responses, despite intact neuronal stimulation. These defects were evident in both microvascular architecture and functional blood-flow measurements, indicating that astrocytic Vps35 is required for translating neuronal activity into vascular growth and perfusion.

At the molecular level, we demonstrate that astrocytic Vps35 is required for proper activation of the HIF1α-VEGFa signaling pathway during postnatal development. HIF1α is a key transcriptional regulator of angiogenesis and is highly active during periods of rapid vascular growth [57, 58]. Using RNAscope, we show that WS induces robust upregulation of Vegfa mRNA specifically in Glast1^+^ astrocytes in control mice. This activity-dependent induction was abolished in Vps35 deficient astrocytes, and WS failed to rescue reduced Vegfa expression in mutants (Fig. 3A, B). These results indicate that astrocytic Vps35 is necessary for enabling astrocytes to elicit an angiogenic transcriptional response to basal and neuronal activity.

To understand why HIF1α levels were reduced in astrocytic Vps35 mutants, we examined PHD co-factors (α-KG and Fe^2+^) that regulate HIF1α stability [59, 60]. Our results showing that α-KG levels were reduced in mutant cortex and primary astrocytes argue against enhanced α-KG driven HIF1α degradation. Instead, we identified iron overload as the primary driver of HIF1α destabilization, because elevated ferrous iron levels in cultured mutant astrocytes and iron deposition in mutant cortex are associated with enhanced HIF1α hydroxylation and degradation in the absence of Vps35 (Figs. 4D-G); and iron chelator treatments in culture and in vivo rescued these phenotypes (Fig. 5). These findings provide a mechanistic link between iron homeostasis and HIF1α-VEGFa signaling.

How does astrocytic VPS35 regulate cellular iron homeostasis? Vps35 is a core component of the retromer complex, which governs endosomal trafficking and recycling of transmembrane proteins [28, 30, 31]. We found that the loss of Vps35 selectively reduced protein levels of FPN, an iron exporter, without altering expression of TFR1, the primary iron import receptor (Figs. 7A, B, E, F). These results suggest that impaired iron export, rather than increased iron uptake, may underlie intracellular iron accumulation. The selective effect on FPN is consistent with retromer-dependent trafficking of iron regulatory proteins from endosomes back to the plasma membrane or the trans-Golgi network. FPN requires continuous retromer-mediated recycling to maintain its surface expression; in the absence of VPS35, FPN is instead mis-trafficked to lysosomes where it undergoes degradation, reducing iron export capacity and driving intracellular iron accumulation [73]. Although TFR1 protein levels were unchanged in our model, impaired retromer-dependent recycling of TFR1 could nevertheless alter its endosomal dwell time and trafficking itinerary, potentially contributing to dysregulated iron flux independent of steady-state protein abundance. Taken together, these findings position VPS35 as a critical node in astrocytic iron homeostasis by maintaining the surface availability of iron export machinery, and implicate endosomal mistrafficking of FPN as the primary mechanism by which retromer loss leads to intracellular iron overload in astrocytes.

We note that conditional loss of Vps35 in astrocytes led to a secondary reduction in VPS26A and VPS29 protein levels, consistent with the well-established interdependence of retromer subunit stability. VPS35 serves as the central scaffold of the VPS35–VPS26–VPS29 heterotrimer, and its loss results in proteasomal degradation of the remaining subunits, a post-translational effect documented across multiple cell types and model systems [51, 76–78]. Importantly, this relationship is asymmetric: VPS26A loss alone does not reciprocally destabilize VPS29 or VPS35, indicating that VPS35 and VPS29 form the primary stable core, and that the secondary reduction in VPS26A reflects complex destabilization rather than an independent contribution from VPS26A itself [51]. Together, these observations support VPS35 loss as the primary driver of the phenotypes described here. Nevertheless, whether astrocyte-specific deletion of VPS26A or VPS29 alone is sufficient to phenocopy the vascular and ferroptosis defects observed in Vps35 KO mice remains an important open question that warrants future investigation.

Beyond its effects on angiogenic signaling, iron overload had profound consequences for astrocyte survival. Excess iron promotes ferroptosis, a regulated form of cell death driven by iron dependent lipid peroxidation [61, 65]. Our results of increased expression of PLIN2, a marker associated with lipid droplet accumulation and oxidative stress, and reduced levels of GPX4, a key enzyme that suppresses ferroptosis by detoxifying lipid hydroperoxides, in mutant astrocytes (Fig. 6A–F) support the view for elevated ferroptosis in Vps35 deficient astrocytes. These molecular changes were present both in vivo and in primary astrocyte cultures, indicating that ferroptotic signaling is cell autonomous.

Iron chelation experiments further support a causal role for iron overload in driving both astrocyte dysfunction and vascular pathology. Treatment with DFO restored HIF1α protein levels, reduced HIF1α hydroxylation, attenuated ferroptosis markers, and improved vascular density and structure in mutant astrocytes or mice (Figs. 5B, C, E, F, G, H; 6C–H). Notably, DFO treatment increased FPN levels despite persistently low Vps35 expression, suggesting that iron chelation can stabilize FPN through retromer-independent mechanisms, likely by relieving iron dependent repression and/or reducing FPN degradation. These findings indicate that iron dysregulation lies upstream of HIF1α destabilization, ferroptosis, and vascular defects, and that targeting iron homeostasis can partially bypass retromer dysfunction.

In addition to astrocytic ferroptosis in the mutant mice, loss of astrocytic Vps35 also impaired morphology and astrocyte maturation. Mutant astrocytes exhibited reduced soma size and decreased process complexity, features associated with immature or dysfunctional astrocytes. Analysis of astrocyte lineage and maturation markers revealed selective alterations rather than wholesale fate changes. While Olig2 expression was unchanged, indicating no aberrant oligodendrocyte lineage adoption, expression of glutamine synthetase, a hallmark of mature astrocytic metabolic function, was reduced in mutants. Increased co-labeling of S100β with GFP suggests an altered astrocytic state, potentially reflecting stress or incomplete maturation. These changes likely compromise astrocyte metabolic support and further exacerbate vascular instability. Beyond the iron-ferroptosis axis, VPS35 loss may impair astrocyte maturation and vascular homeostasis through additional mechanisms involving BMP signaling and its downstream effectors. BMP signaling via Smad1/5/8 phosphorylation is a well-established driver of astrocyte maturation, promoting the acquisition of mature morphological and molecular identity including process elaboration, GS expression, and S100β immunoreactivity [79, 80]. A critical co-receptor in this pathway is Neogenin, a transmembrane member of the DCC family required for BMP2-dependent activation of YAP and sustained Smad1/5/8 signaling in neural stem cells and neocortical astrocytes; loss of Neogenin impairs BMP2-induced astrogliogenesis and astrocyte maturation in vivo [81]. Beyond maturation, astrocytic Neogenin plays a direct role in cortical blood vessel homeostasis. Astrocyte-specific Neogenin knockout mice exhibit leaky vessels, reduced blood flow, disrupted vascular basement membranes, and reduced pericyte coverage, phenotypes mediated through Neogenin-dependent BMP induction of netrin-1 in astrocytes [82]. These observations are notable in light of our findings, as the vascular and maturation deficits in Vps35KO mice share features with those reported in astrocyte-specific Neogenin mutants. Whether VPS35 loss impairs BMP–Neogenin signaling in astrocytes, through mechanisms yet to be determined, and thereby contributes to both the maturation deficits and vascular abnormalities we observe, represents an important question for future investigation.

The mechanisms uncovered here may have broader relevance to neurodegenerative diseases characterized by retromer dysfunction, iron accumulation, and vascular pathology. Reduced Vps35 expression and impaired retromer function have been reported in AD, PD, and ADRD, conditions that also exhibit cerebrovascular dysfunction and disrupted iron homeostasis [61, 83]. Astrocytes are increasingly recognized as central regulators of iron buffering and redistribution in the brain, and failure of astrocytic iron handling can amplify oxidative stress, neuronal vulnerability, and vascular instability. Our findings suggest that astrocytic retromer dysfunction may represent an upstream driver linking intracellular trafficking defects to ferroptosis, astrocyte loss, and neurovascular compromise in disease contexts.

In summary, we propose a model (Fig. 7K) in which astrocytic Vps35 or the retromer complex maintains FPN dependent iron export to prevent intracellular iron overload. Loss of Vps35 leads to cellular iron accumulation, which enhanced not only HIF1α hydroxylation and degradation, leading to suppression of VEGFa signaling and impairing basal and activity-dependent angiogenesis, but also ferroptosis, resulting in astrocyte loss. By connecting endosomal trafficking to iron metabolism, astrocyte and neurovascular development, this study identifies astrocytic Vps35 as a critical integrator of iron metabolism and vascular homeostasis in the developing cortex and highlights iron regulation as a potential therapeutic target in retromer associated pathologies.

## Materials and methods

### Animals

Mice were maintained according to animal protocols approved by Case Western Reserve University (CWRU) according to the National Institute of Health (NIH) guidelines. All mice were housed in standard conditions with food and water provided and a 12 h dark/light cycle. Vps35-floxed (Vps35^f/f^) mice were generated, maintained, and genotyped as described previously [84]. GFAP-Cre mice (stock 024098) [86] https://doi.org/10.1523/JNEUROSCI.3095-08.2009, GFAP-Cre/ERT2 (stock 012849) [87] https://doi.org/10.1523/JNEUROSCI.2532-06.2006, and RCL-EYFP (Ai3) (stock 007903)https://doi.org/10.1038/nn.2467 [88] were purchased from Jackson Laboratories. The Vps35^f/f^ mutant mice were crossed with GFAP-Cre and GFAP-CreER^T2^ mice to generate Vps35^GFAP−Cre^ and Vps35^GFAPcreER^, respectively. Ai3 mice were crossed with Vps35^GFAPcreER^ and GFAP-CreER^T2^ mice to generate Vps35^GFAPcreER^;Ai3 and GFAP-CreER^T2^;Ai3, respectively. Mice tail samples were genotyped using strain-specific primers through PCR to amplify targeted DNA sequences to identify transgenic and knockout alleles. GFAP-CreER^T2^ Primers F: GGTCGATGCAACGAGTGATGAGG R: GCTAAGTGCCTTCTCTACACCTGCG Vps35^f/f^ Primers: F: AACCAGCTCCCAACAAAATG R: GCTTGGTCCCACTCACATTT. GFAP-Cre Primers: F: TCCATAAAGGCCCTGACTC R: TGCGAACCTCACATCGT. All the mouse lines indicated above were maintained in a C57BL/6 background for more than six generations. Male and female mice were examined throughout all the experiments.

### Antibodies

The following antibodies were used: rabbit polyclonal anti-Vps35 antibody was generated against the murine Vps35 C-terminal sequence as described previously [42]; rabbit polyclonal anti-Vps26a (Abcam, 23,892); mouse monoclonal anti-Vps29 (Santa Cruz, sc-398874); rabbit monoclonal anti-HIF1α (Cell Signaling Technology, 3716S); rabbit monoclonal anti-HIF1α-OH (Cell Signaling Technology, 3434S); rabbit polyclonal anti-PLIN2 (Proteintech, 15,294–1-AP); rabbit monoclonal anti-GPX4 (Abcam, 125,066); mouse monoclonal anti-TfR1/CD71 (Invitrogen, 13–6800); rabbit polyclonal anti-FPN (Thermofisher, PA5-77,470); mouse monoclonal anti-β-Actin (Cell Signaling, 3700S); rat monoclonal anti-PODXL (R&D Systems, 192,703); goat polyclonal Iba1 (Abcam, ab5076); rabbit monoclonal anti-GFAP (Cell Signaling, 12,389); mouse polyclonal anti-cleaved caspase-3 (Invitrogen, 16,843); mouse monoclonal anti-CD31/PECAM (Cell Signaling, 3528); monoclonal anti-SMA-647 (Santa Cruz Biotechnology, sc-32251); rat monoclonal anti-Ki67(Synaptic Systems, HS-398 117); mouse monoclonal anti-Laminin-α5 (Abcam, ab17107); chicken polyclonal anti-GFP (Aves Labs, GFP-1020); mouse monoclonal anti-Olig2 (EMD Millipore, MABN50); guinea-pig polyclonal anti-S100β (Synaptic Systems, 287,004); and mouse monoclonal anti-GS (Sigma, MAB302).

### Tamoxifen and DFO treatment

Vps35^GFAPcreER^;Ai3 and GFAP-CreER^T2^;Ai3 mice received intraperitoneal (i.p.) injections of tamoxifen (75 mg/kg of tamoxifen; 10 mg/ml Sigma Millipore, catalog #T5648) dissolved in corn oil (Sigma Millipore, catalog #C8267) and ethanol at a 9:1 ratio. Injections were administered once daily at postnatal day 7 (P7) for three consecutive days to induce Cre-mediated recombination under the GFAP promoter. Mice treated with deferoxamine (DFO; 100 mg/kg; Sigma-Millipore catolog #252,750) dissolved in 0.9% NaCl and received daily I.P. injections of DFO from P11 to P21.

### Tissue processing and immunostaining

Mice of the indicated genotypes were anesthetized and perfused transcardially with phosphate-buffered saline (PBS; 0.01 M, pH 7.4), followed by 4% paraformaldehyde (PFA; Germantown, WI, USA; pH 7.4). Brains were removed, post-fixed overnight in 4% PFA at 4 °C, and sectioned coronally at 40 µm thickness using a vibratome (Leica VT1000S). Free-floating sections were collected in PBS and stored at − 20 °C in cryoprotectant solution (FD Section Storage Solution) until further processing. Meningeal tissue isolation was performed as previously described [53]. For staining, sections were washed three times in PBST (PBS containing 0.05% Triton X-100; 5 min each) and blocked for 1 h at room temperature in PBST supplemented with 3% donkey serum. Samples were then incubated overnight at 4 °C with primary antibodies diluted in PBST. The following day, sections were washed three times in PBST and incubated with Alexa Fluor–conjugated secondary antibodies (1:300; Thermo Fisher Scientific) for 2 h at room temperature. After final washes, sections were counterstained with DAPI (Thermo Fisher Scientific) to visualize nuclei. Stained sections were mounted using SlowFade™ Diamond Antifade Mountant (Thermo Fisher Scientific; Cat# S36972) and imaged using a Zeiss LSM 800 confocal microscope.

### RNAscope: fluorescent multiplex in-situ hybridization

RNAscope analysis was conducted on mouse brain tissue using the RNAscope® Multiplex Fluorescent Detection Kit (Advanced Cell Diagnostics, Noble Park North, Australia; Cat# 323,110) following the manufacturer’s instructions. The probes used included *Mm-Slc1a3/GLAST* (Cat# 430,781) and *Mm-VEGFa-O1* (Cat# 21300A), and all hybridization steps were performed in a HybEZ™ oven (Cat# 321,711; ACD, Inc.). Mice were anesthetized and transcardially perfused with 4% paraformaldehyde (PFA, pH 7.4) at postnatal day 21 (P21). Brains were post-fixed in 4% PFA for 16 h at 4 °C, cryoprotected in 15% and 30% sucrose solutions, and sectioned at 14 µm thickness using a freezing microtome (Leica, Buffalo Grove, IL, USA). Sections were dehydrated through graded ethanol and treated with hydrogen peroxide to quench endogenous peroxidase activity. RNA retrieval was performed by incubating the sections in pre-warmed retrieval buffer at 100–103 °C for 15 min, followed by rinsing in deionized water. Hybridization was carried out using both negative (bacterial gene) and positive (*Polr2a*, RNA polymerase II) control probes for 2 h at 40 °C. After washing, the slides were sequentially incubated with preamplifier and amplifier solutions for 30 and 15 min, respectively, at 40 °C. Fluorescent labeling was achieved using the Opal™ Multiplex System (AKOYA Biosciences; Cat# NEL810001KT) with Opal 520 (green), Opal 570 (red), and Opal 690 (far-red) fluorophores. Nuclei were counterstained with DAPI, and fluorescent images were acquired using a confocal microscope.

### Whisker stimulation

Whisker stimulation was performed as previously described [52]. Briefly, mouse pups underwent daily whisker stimulation from postnatal day 14 (P14) to P21. During each session, pups were gently restrained on a metal cylinder, and the right-side whiskers were manually brushed at a frequency of 3–4 Hz for 15 min using a Fitchew brush. Mice were sacrificed 90 min after the final stimulation session.

### Western blotting

Tissues were dissected and homogenized in ice-cold RIPA buffer (50 mM Tris–HCl, pH 7.4; 150 mM NaCl; 2 mM EDTA; 1 mM PMSF; 50 mM NaF; 1 mM Na₃VO₄; 1% sodium deoxycholate; and 1% SDS) supplemented with a complete protease inhibitor cocktail (Roche, Cat# 04693159001). The homogenates were centrifuged at 13,000 × g for 15 min at 4 °C, and supernatants were collected for protein analysis. Protein concentrations were determined using the BCA Protein Assay Kit (Pierce Biotechnology). Equal amounts of protein were mixed with loading buffer, separated on 9% or 11% SDS–PAGE gels, and transferred to nitrocellulose membranes using a wet-transfer apparatus at 30 V overnight. Membranes were blocked with 3% bovine serum albumin (BSA) in TBS containing 1% Triton X-100 (TBST) and washed three times for 10 min each with TBST. Blots were incubated overnight at 4 °C with primary antibodies diluted in 5% BSA/TBST, followed by three washes and incubation with species-specific HRP-conjugated secondary antibodies (1:5000, Thermo Fisher). Protein signals were visualized using enhanced chemiluminescence (ECL, Pierce, Rockford), and images were captured with an Odyssey Fc imaging system (LI-COR). Band intensities were quantified using ImageJ, and protein expression was normalized to β-actin as described previously [89] https://doi.org/10.1016/j.biopsych.2020.08.026. 

### Primary astrocyte culture

Primary astrocytes were isolated from neonatal Vps35^GFAP−Cre^ mice and control mice as previously described [81, 82]. Briefly, cerebral cortices from postnatal day 1–3 (P1–P3) pups were dissected free of meninges, minced into small fragments, and incubated in 0.125% trypsin at 37 °C for 20 min. The digested tissue was then mechanically dissociated into a single-cell suspension using 40 µm cell strainers. Cells were plated onto poly-L-lysine–coated (0.1 mg/ml; Sigma) culture dishes and maintained in DMEM supplemented with 10% fetal bovine serum (FBS; Invitrogen) at 37 °C in a humidified 5% CO_2_ incubator. After 6–8 days in culture, when astrocytes reached confluence, microglia and oligodendrocyte precursor cells (OPCs) were removed by shaking the cultures at 200 rpm overnight. Astrocytes were then detached using 0.25% trypsin–EDTA (Invitrogen), and the supernatant was discarded. The remaining confluent astrocyte layer was rinsed twice with PBS, incubated with 5 ml of trypsin–EDTA at 37 °C, and subsequently collected for downstream experiments. The purity of glial fibrillary acidic protein (GFAP)–positive astrocytes in this culture system was greater than 95%.

### Measurements of iron and α-KG

**α‐KG detection**: α-KG levels were determined using the Alpha-Ketoglutarate Assay Kit (Abcam; Cat# ab83431) according to the manufacturer’s protocol. A standard curve was generated from α-KG standards. Cell samples were suspended in α-KG assay buffer and centrifuged at 13,000 × g for 5 min at 4 °C. The supernatant was transferred to a clean tube, and proteins were precipitated with perchloric acid. The pH was adjusted to 7.5 with KOH, followed by centrifugation at 13,000 × g for 15 min at 4 °C. The final supernatant was used for detection. To quantify α-KG, the α-KG converting enzyme, enzyme mix, and colorimetric probe were added to each well of a 96-well plate containing assay buffer. The mixture was incubated for 30 min at 37 °C in the dark, and absorbance was measured at 570 nm using a spectrophotometer.

**Total Iron assay**: Cellular ferrous iron (Fe^2^⁺) levels were measured using the Iron Assay Kit (Sigma-Aldrich; Cat# MAK025) following the manufacturer’s protocol. Briefly, tissue or cell lysates were homogenized in 4–10 volumes of iron assay buffer and centrifuged at 16,000 × g for 10 min at 4 °C to remove insoluble material. The resulting supernatant was collected, and 50 µL of each sample was added to wells of a 96-well plate, adjusted to a final volume of 100 µL with assay buffer. After incubation at 37 °C for 30 min, absorbance was measured at 593 nm using a microplate reader. Relative Fe^2^⁺ concentrations were calculated from a standard curve and normalized to total protein content.

**Iron Stain:** Iron accumulation in brain tissue was assessed using the Prussian Blue Iron Stain Kit (Abcam; Cat# ab150674) following the manufacturer’s protocol and previous publication [85]. Fresh-frozen brain samples were sectioned at 14 µm thickness using a freezing microtome (Leica, Buffalo Grove, IL, USA). Sections were incubated in a 1:1 mixture of potassium ferrocyanide and hydrochloric acid solutions to facilitate the formation of ferric ferrocyanide complexes. After rinsing in distilled water, sections were counterstained with Nuclear Fast Red, washed, and dehydrated through an ethanol series. The resulting blue precipitate indicated ferric iron deposition. Stained sections were visualized and imaged using a Zeiss LSM 800 confocal microscope.

### Cerebral cortical blood flow in mouse cortex by Laser Speckle Contrast Imaging (LSCI)

Mice were anesthetized with isoflurane and positioned in a digital stereotaxic apparatus. The skull and mucous membrane (~ 1.76 cm^2^ area) were exposed for blood perfusion measurement using a laser speckle contrast imaging (LSCI) system (RWD Life Science) with a scanning depth of 1 mm. The LSCI system was focused on the skull surface to obtain a clear color map. Normal saline was applied to the skull throughout the recording to maintain a wet condition. Cortical blood flow from both cerebral hemispheres was recorded for 5 min each. The average perfusion unit (PU) across the recorded time was quantified and compared between groups.

### Real time PCR analysis

Total RNA was isolated from the primary somatosensory cortex or cultured primary astrocytes (three mice per group) using TRIzol reagent (Invitrogen). A total of 5 µg of purified RNA was reverse-transcribed into complementary DNA (cDNA) using the GoScript™ Reverse Transcription System (Promega). The synthesized cDNA was then used for quantitative real-time PCR (qPCR) with SYBR Green Master Mix (QIAGEN) on a CFX96 Real-Time PCR Detection System (Bio-Rad). Each reaction was performed in triplicate, and gene expression levels were normalized to *Gapdh* using the 2^−ΔΔCt^ method. Primer sequences used for amplification were as follows: Vps35 (forward: 5′-GACTT CGCTG ATGAA CAGAG CC-3′, reverse: 5′-TCAGC GGATT CGCTT CACAC TG-3′); VEGFa (forward: 5′-GCAGG CTGCT GTAAC GATGA-3′, reverse: 5′-TCATG CGGAT CAAAC CTCAC-3′); VEGFc (forward: 5′-CCTGA ATCCT GGGAA ATGTG CC -3′, reverse: 5′-CGATT CGCAC ACGGT CTTCT GT -3′); and GAPDH (forward: 5′-CATCA CTGCC ACCCA GAAGA CTG-3′, reverse: 5′-ATGCC AGTGA GCTTC CCGTT CAG-3′).

### Cell counting and average intensity plots

Confocal images were acquired using a 20 × objective with 2-µm optical steps and 0.5 × digital zoom. Maximum-intensity Z-projections were generated from ten consecutive optical sections for all analyses. Quantification of cell number was performed in ImageJ (NIH) using the Cell Counter plugin by identifying either blood vessel–marker–positive structures or nuclei positive for cell-type–specific markers co-localized with DAPI within a defined region of interest (ROI; layers I–VI of the primary somatosensory cortex). All ROIs were matched in area and volume to ensure equivalent representation of DAPI-labeled nuclei across samples. Fluorescence intensity measurements were obtained from the same ROIs. Expression density for each marker (e.g., CD31 or PODXL) was calculated by normalizing the integrated intensity to DAPI signal and dividing by the ROI area. Average intensity profiles were generated using the Plot Profile function in ImageJ. For line-scan analyses, pixel values were averaged in 10-µm bins across the ROI. For laminar analyses, intensity values were extracted and averaged for each cortical layer. All image processing and quantifications were performed using identical acquisition parameters and analysis settings across experimental groups.

### Statistical analysis

All data were presented as mean ± SD. For immunohistochemical analyses, three or more brain slices from at least three mice per group were examined, and for in vitro assays, data were obtained from a minimum of three independent experiments. Quantification of immunofluorescence and western blot data was performed using ImageJ software. Blood vessel parameters, including density, length, branching, and thickness, were analyzed using the Vessel Analysis package. Statistical analyses were conducted using Prism 10 (GraphPad Software). Comparisons between two groups were made using an unpaired Student’s *t*-test, whereas one-way analysis of variance (ANOVA) followed by Tukey’s post hoc test was used for comparisons among three or more groups. The specific *n* values were indicated in the figure legends. Statistical significance was defined as *P* < 0.05.

## Supplementary Information

Below is the link to the electronic supplementary material.

Supplementary file1.

## Data Availability

All data needed to evaluate the conclusions in the paper are present in the paper and/ or the supplementary information. Data will be made available on reasonable request.

## Abbreviations

AD: Alzheimer’s disease
ADRD: Alzheimer’s disease related dementia
ALS: Amyotrophic lateral sclerosis
BBB: Blood-brain barrier
BV: Blood vessel
c-Caps-3: Cleaved caspase-3
CD31(PECAM-1): Platelet endothelial cell adhesion molecule-1
DFO: Deferoxamine
EC: Endothelial cell
FPN: Ferroportin
GFAP: Glial fibrillary acidic protein
GFP: Green fluorescent protein
GPX4: Glutathione peroxidase 4
GS: Glutamine synthetase
HIF1α-OH: Hydroxylated hypoxia-inducible factor 1-alpha
HIF1α: Hypoxia-inducible factor 1-alpha
IBA1: Ionized calcium-binding adapter molecule 1
KI67: Antigen kiel 67
LAMα5: Laminin-α5
mRNA: Messenger RNA
NS: Non-stimulated
Olig2: Oligodendrocyte transcription factor 2
PD: Parkinson’s disease
PHD: Prolyl hydroxylase domain
PLIN2: Perilipin 2
PODXL: Podocalyxin-like
RT-qPCR: Real-time PCR
S100β: S100 calcium-binding protein B
SMA: Smooth muscle actin
TAM: Tamoxifen
TFR1: Transferrin receptor 1
TGF-β: Transforming growth factor-beta
VEGFa: Vascular endothelial growth factor a
VPS35: Vacuolar protein sorting 35
WS: Whisker stimulation
α-KG: α-Ketoglutarate
