# Supplementary material for: Astrocytic VPS35 coordinates iron homeostasis with HIF1α-VEGFa signaling to prevent astrocytic ferroptosis and to promote neocortical angiogenesis
Source: Cell Biosci. 2026 Jun 24;16:106. doi: 10.1186/s13578-026-01602-2 (PMC13563901; doi:10.1186/s13578-026-01602-2)
Supplement: Supplementary file 1 [file 13578_2026_1602_MOESM1_ESM.docx]

**Supplemental Figure legends**

**Supplemental Fig. 1. Generation of astrocyte specific Vps35 knockout mice**

(A) Schematic of the animal breeding to generate astrocyte-specific Vps35 knockout mice. (B) Representative immunoblots of S1 cortex (ctx) homogenates from P21 control (Vps35^f/f^) and astrocyte-specific Vps35 knockout (Vps35^GFAPcreER^) mice using indicated antibodies. (C) Quantification of VPS35, VPS26A, and VPS29 protein levels shown in (B). Data are presented as mean ± SD; n = 3 mice per group; unpaired Student’s t-test (*p < 0.05, **p < 0.01). (D) Representative RNAscope images showing Glast1 mRNA (green), Vps35 mRNA (red), and DAPI (blue) in S1 cortex (layer I-II) from P21 control and Vps35^GFAPcreER^ mice. Scale bar: 20 µm. (E) Quantification of Vps35 mRNA intensity in Glast1^+^ astrocytes and Glast1^-^ cells from data in (D). Data are presented as mean ± SD; n = 3 mice per group; unpaired Student’s t-test (**p < 0.01). (F) Body-weight growth curves of control and Vps35^GFAPcreER^ mice at ages of P7 to P21 (n = 10–15 mice per group).

**Supplemental Fig. 2. Hippocampal vascular density and diameter is reduced in astrocyte Vps35-KO mice**

(A) Representative immunostaining images of blood vessels (BVs) in the hippocampus labeled with PODXL⁺ (magenta) and DAPI (blue) in control and Vps35^GFAPcreER^ mice. (B) Quantification of PODXL⁺ BV density (#/mm³). (C) Quantification of PODXL⁺ BV vessel diameter (µm). Quantification of PODXL⁺ BV branch number (#/mm³). Data are presented as mean ± SD; n = 4-5 mice per group; unpaired Student’s t-test (*p < 0.05). Scale bar: 100 µm.

**Supplemental Fig. 3. Loss of astrocytic Vps35 disrupts maintenance of cerebrovascular density in adult mice**

(A) Schematic of the experimental design. Vps35^f/f^ and Vps35^GFAPcreER^ mice were treated with tamoxifen (75 mg/kg, intraperitoneally) for three consecutive days at 1 month of age and analyzed at 2 months. (B) Representative immunostaining images of CD31⁺ BVs in the S1 cortex. (C–F) Quantification of PODXL⁺ BV density (C), vessel length (D), branch number (E), and vessel diameter (F). Data are presented as mean ± SD; n = 3 mice per group; unpaired Student’s t-test (*p < 0.05, **p < 0.01). Scale bar: 50 µm.

**Supplementary Fig. 4. Reduced meningeal blood-vessel density and diameter in Vps35^GFAPcreER^ mice**

(A) Schematic illustrating whole-mount transverse tissue sectioning for immunostaining of cortical surface vasculature. (B) Representative immunostaining images of PODXL⁺ BVs (green) and Iba1⁺ microglia (red) in meningeal regions. (C) Quantification of PODXL⁺ BV density and Iba1⁺ cell density (#/mm³). (D) Representative immunostaining images of smooth muscle actin (SMA)-positive arteries. (E) Quantification of SMA⁺ arterial density (#/mm³) and arterial diameter (µm). Data are presented as mean ± SD; n = 3 mice per group; unpaired Student’s t-test (*p < 0.05, **p < 0.01). Scale bar: 50 µm.

**Supplementary Fig. 5. Altered astrocyte morphology in Vps35^GFAPcreER^ cortex**

(A) Representative high-magnification images of GFP⁺ astrocytes from GFAPcreER;Ai3 control and Vps35^GFAPcreER^;Ai3 mice, with three-dimensional reconstructions generated using Imaris software. (B) Quantification of astrocyte soma area and branch length. Data are presented as mean ± SD; n = 50–60 cells from 3 mice per group; unpaired Student’s t-test (**p < 0.01).

**Supplementary Fig. 6. Altered maturation in Vps35 deficient astrocytes**

(A) Representative immunostaining images of S100B⁺ GFP⁺ astrocytes from GFAPcreER;Ai3 control and Vps35^GFAPcreER^;Ai3 mice. (B) Quantification of S100B⁺ cell density and percentage associated with GFP⁺ astrocytes. (C) Representative immunostaining images of OLIG2⁺ GFP⁺ cells. (D) Quantification of OLIG2⁺ cell density and percentage associated with GFP⁺ astrocytes. (E) Representative immunostaining images of GS⁺ GFP⁺ cells. (F) Quantification of GS⁺ cell density and percentage associated with GFP⁺ astrocytes. Data are presented as mean ± SD; n = 4–6 mice per group; unpaired Student’s t-test (*p < 0.05). Scale bar: 40 µm.

**Supplemental figure 1. Generation of astrocyte specific Vps35 knockout mice**

**Supplemental figure 2. Hippocampal vascular density and diameter is reduced in astrocyte Vps35-KO mice**

**Supplemental figure 3. Loss of astrocytic Vps35 disrupts maintenance of cerebrovascular density in adult mice**

**Supplemental Figure 4. Reduced meningeal blood-vessel density and diameter in Vps35^GFAPcreER^ mice**

**Supplemental Figure 5. Altered astrocyte morphology in Vps35^GFAPcreER^ cortex**

**Supplemental Figure 6. Altered maturation in Vps35 deficient astrocytes**
